# The Nurse as Health Visitor

**Published:** 1920-07-24

**Authors:** 


### THE MATRONS' AND SISTERS' DEPARTMENT.

### THE NURSE AS HEALTH VISITOR.

The grievance of nurses in respect to the profession of health visiting has often been alluded to in our columns. When as a highly qualified public servant the nurse leaves her training-school after three, four, or more years spent in mastering the problems which affect the health of the public, she finds herself confronted with the necessity for spending an additional year in study, during which time she must maintain herself. Even after this she finds girls competing with her for appointments who have merely put in a couple of years' training. The trained nurse is discouraged from qualifying as a health visitor by the prohibitive cost of the extra year's college training, and by the long postponement in the commencement of her career which it entails. But far, far more is the prospective health visitor discouraged by these impediments from using the nurse training-schools as the natural portal to her career. She counts the cost; she reckons out the time, and she takes another route to her goal. More and more health visiting is being undertaken by women who have only a smattering of nursing lore in addition to their " health " certificates.

The College of Nursing, we are greatly interested to learn from the last *Bulletin*, has tackled both these problems—that of the trained nurse who wants to become a health visitor but cannot afford to keep herself for a year while in training, and that of the woman who would rather enter the profession of health visiting by the right portal of trained nursing, but shirks the issue because, after becoming a trained nurse, she will still be unqualified for the career.

The position of the trained nurse who desires to qualify as a health visitor has been brought before the Board of Education by the College, and has received sympathetic consideration. An assurance has been given that a limited number of experimental grants will be made to certificated nurses who desire to enter upon a period of training for the post of health visitor. The importance of this announcement will be readily apprehended. It removes the great bar which holds back women on the completion of their training from qualifying for these interesting appointments, and it will pave the way for the entry of many able women into a profession which is crying out for such candidates. We trust before long to announce the particulars under which these grants will be made available. And meantime it would be well if the heads of training-schools would bear in mind the coming scholarships, for such in effect they will be, and would make probationers who will be concluding their course this autumn aware of them.

The scholarship system is, however, rather of the nature of a temporary expedient than a permanent remedy for the health-visiting impasse. A further step, the co-operation of the hospitals in the development of the ideal health visitor, is necessary if the trained nurse is to be put on a level of opportunity with the lay woman. The *Bulletin* announces that " the College has ascertained that some nurse training-schools are willing immediately to modify their curriculum in such a way as to include the subjects laid down in the Board's regulations for the training of health visitors, and has received an assurance that these regulations, now being redrafted, will make such schemes as have been suggested possible. The effect of this action on the part of the training-schools in question will be to introduce into the course of probationers who are looking forward to becoming health visitors instruction in preventive work. Some hospitals are able to offer fourth-year students a period of practical work in their maternity and child-welfare centres. By a little rearrangement of the curriculum it should be possible for the training-schools to satisfy the Board of Education that they are capable of preparing their students from start to finish, direct from hospital, to enter for such examinations as may be prescribed. In this way an incentive will be given to students desiring the best possible qualification to take it through the nurse training-schools.

There has never been the least doubt in the minds of most medical officers of health under whom health visitors are called to work, that thorough training in hospital is the best form of preparation for this career. In some towns the highest posts are only open to trained nurses. In many trained nurses secure a higher rate of pay. Once remove the disabilities under which nurses labour in entering this profession and it may be anticipated that certificated nurses will oust lay candidates from the field by demonstrating the supreme advantage of hospital training.

